# Nomogram for Predicting Lymph Node Involvement in Triple-Negative Breast Cancer

**DOI:** 10.3389/fonc.2020.608334

**Published:** 2020-12-04

**Authors:** Xiang Cui, Hao Zhu, Jisheng Huang

**Affiliations:** Department of Thyroid and Breast Surgery, The First People’s Hospital of Shangqiu, Shangqiu, China

**Keywords:** lymph node involvement, triple-negative breast cancer, nomogram, prediction, Surveillance, Epidemiology and End Results

## Abstract

**Background:**

Lymph node metastasis of triple-negative breast cancer (TNBC) is essential in treatment strategy formulation. This study aimed to build a nomogram that predicts lymph node metastasis in patients with TNBC.

**Materials and Methods:**

A total of 28,966 TNBC patients diagnosed from 2010 to 2017 in the Surveillance, Epidemiology and End Results (SEER) database were enrolled, and randomized 1:1 into the training and validation sets, respectively. Univariate and multivariate logistic regression analysis were applied to identify the predictive factors, which composed the nomogram. The receiver operating characteristic curves showed the efficacy of the nomogram.

**Result:**

Multivariate logistic regression analyses revealed that age, race, tumor size, tumor primary site, and pathological grade were independent predictive factors of lymph node status. Integrating these independent predictive factors, a nomogram was successfully developed for predicting lymph node status, and further validated in the validation set. The areas under the receiver operating characteristic curves of the nomogram in the training and validation sets were 0.684 and 0.689 respectively, showing a satisfactory performance.

**Conclusion:**

We constructed a nomogram to predict the lymph node status in TNBC patients. After further validation in additional large cohorts, the nomogram developed here would do better in predicting, providing more information for staging and treatment, and enabling tailored treatment in TNBC patients.

## Introduction

Breast cancer, the most common malignant tumor in women, is a heterogeneous disease. Triple-negative breast cancer (TNBC) represents one of the subtypes described in recent years, which does not express estrogen receptor (ER), progesterone receptor (PR) or human epidermal growth factor receptor 2 (HER2). It shows a variety of biological, clinicopathological and molecular characteristics, responses significantly differently to treatment and achieves divergent prognosis (1, 2). Despite the low incidence, accounting for about 10 to 20% of all breast cancer cases, TNBC shows strong invasiveness, high malignancy and short relapse-free survival, reflecting the vital role of early diagnosis and accurate staging (3). Compared with other subtypes, patients with TNBC are more likely to show lymph node metastasis at the initial diagnosis (4).

Studies have shown that lymph node status is crucial for prognosis prediction and treatment decision in TNBC (5–7). At present, sentinel lymph node biopsy (SLNB), axillary lymph node dissection (ALND) and subsequent pathological diagnosis are commonly used methods to evaluate lymph node status in TNBC. The false negative rate of SLNB is 5–10%, which may result in improper patient management. Sufficient ALND can effectively reduce the risk of TNBC metastasis, but may cause chronic side effects such as numbness, stiffness in the upper body, and lymphedema. Moreover, extra-axillary lymph node metastasis also occurs (8), implying that SLNB or ALND might not be sufficient for the diagnosis of lymph node metastasis in TNBC. Therefore, it is helpful to classify TNBC cases preoperatively based on clinicopathologic factors, which contributes to the development of individualized surgical treatments and reducing overall mortality and morbidity in TNBC.

Clinical researchers and clinicians always make unremitting effort in predicting lymph node (LN) status. Several studies have developed multiple models for LN status prediction, but mostly are based on limited cases (9). Tan et al. constructed an immune-related genes (IRGS)-based nomogram to accurately estimate the preoperative ALN status of 214 operable TNBC cases (10). Despite its strong performance, the gene-based model may be difficult to promote. Therefore, this study aimed to develop a risk nomogram based on clinical data to determine lymph node metastasis, which could help to identify TNBC patients with positive lymph nodes more quickly.

## Materials and Methods

### Patients

We extracted the data of 28,966 triple-negative breast cancer patients registered between January 1, 2010 and December 31, 2017 from the SEER program. HER2 status was absent in SEER’s breast cancer cohort before 2010, and an enormous number of patients diagnosed before this time point were not included. Analysis cohorts were identified according to the following criteria: unilateral, invasive carcinoma of the breast (ICD-O-3 8500); diagnosis confirmed by positive histology and not by autopsy or a death certificate, as the first and only primary tumor; adjusted AJCC stage I–III; known tumor size; histological grade I–III; known regional lymph node status; ER, PR, HER2 negative. Patients with Paget’s disease or younger than 18 years old were excluded. The patients were randomized 1:1 to the training and validation sets, respectively, for the construction and verification the nomogram. The following information was collected and transformed into categorical variables: age, race, gender, laterality, grade, location, histological type, and T stage.

### Construction and Validation of the Nomogram

Lymph node status was determined according to the Regional Nodes Positive term. We first screened the lymph node status-related clinicopathological characteristics, and found that statistically significant variables included age, race, grade, location, histological type, and T stage (P<0.05). All these variables were analyzed by univariate logistic regression analysis, and the correlated ones (P<0.05) are estimated through multivariate logistic regression analysis. As a result, significantly independent predictors were identified to construct a well-calibrated nomogram. Odds ratios (ORs) and 95% confidence intervals (CIs) were also calculated. Nomogram performance was quantified with respect to calibration and discrimination. Calibration was assessed graphically by plotting the relationship between actual (observed) and predicted probabilities by the Hosmer goodness-of-fit test (11). Internal validation of performance was estimated by the bootstrapping method (1,000 replications). According to the nomogram, total points for all patients were determined with the “nomogramFormula” package in the R software. Discrimination (ability of a nomogram to separate patients with different lymph node statuses) was quantified by the area under the receiver operating characteristic (ROC) curve (AUC). A larger AUC (range 0.5–1.0) reflected a more accurate prediction.

Finally, the best cut-off value is determined by the Youden’s index, according to which, the training and validation cohorts were divided into two subgroups. The correlation between the nomogram and the risk of lymph node metastasis was estimated by univariate logistic regression analysis.

### Statistical Analyses

The chi-square test was performed to evaluate the associations of lymph node status with appropriate variables. Fisher’s exact test was carried out if necessary. Statistically significant was defined as two-sided P<0.05 was considered, unless otherwise stated. All statistical analyses were performed using STATA (version 14.1) and R (version 3.6.1). The R packages caret, rms, pROC, ggplot2, parallel, and nomogramFormula were applied.

## Results

### Patient Characteristics

There were 28,966 patients enrolled in this study, with 8,710 (30.07%) lymph node positive (**Table 1**). The demographics and clinicopathologic characteristics related to lymph node status included age, race, grade, location, histological type and T stage. Younger patients (age<60) have a higher rate of lymph node involvement (32.43%) compared with older ones (age≥60, 26.98%) (*P* < 0.001). As for race, 33.79% black patients had positive lymph nodes versus 29.00% for white patients and 30.00% for others (*P* < 0.001). The positive rate of lymph nodes was higher in patients with grade III cancer than grade II and grade I (31.33% vs. 25.88% and 12.20%, respectively; *P* < 0.001). Patients with primary tumor located in the axillary tail of the breast were more likely to have positive lymph nodes (46.26%), while cases primarily located in the central portion of the breast ranked second (39.06%) (*P* < 0.001). Patients with invasive lobular carcinoma (ILC) (44.05%) had higher positive rate of lymph nodes than invasive ductal carcinoma (IDC), IDC/ILC, and other histological types (30.43%, 43.38% and 24.78%, respectively) (*P* < 0.001). It was found that lymph nodes are positive correlated with T stage. Stage T4 cases had the highest rate of positive lymph nodes (68.48%), versus T1, T2, and T3 patients (18.23%, 34.97% and 53.18%, respectively) (*P* < 0.001) (**Table 1**).

**Table 1:** Patients’ demographics and clinicopathologic characteristics by lymph node status.

	Whole cohort				Training cohort				Validation cohort			
	LN- (%)	LN+ (%)	Total	P	LN- (%)	LN+ (%)	Total	P	LN- (%)	LN+ (%)	Total	P
**All**	20,256 (69.93)	8,710 (30.07)	28,966		10,142 (70.03)	4,341 (29.97)	14,483		10,114 (69.83)	4369 (30.17)	14,483	
**Age**				**<0.001**				**<0.001**				**<0.001**
<60	11,094 (67.57)	5,325 (32.43)	16,419		5,493 (67.37)	2,661 (32.63)	8,154		5,601 (67.77)	2,664 (32.23)	8,265	
≥60	9,162 (73.02)	3,385 (26.98)	12,547		4,649 (73.46)	1,680 (26.54)	6,329		4,513 (72.58)	1,705 (27.42)	6218	
**Race**				**<0.001**				**<0.001**				**<0.001**
White	14,666 (71.00)	5,990 (29.00)	20,656		7,341 (71.20)	2,970 (28.8)	10,311		7,325 (70.81)	3,020 (29.19)	10,345	
Black	3,968 (66.21)	2,025 (33.79)	5,993		1,987 (65.59)	1,038 (34.31)	3,025		1,981 (66.75)	987 (30.94)	2,968	
Others^#^	1,622 (70.00)	695 (30.00)	2,317		814 (70.97)	333 (29.03)	1,147		808 (69.05)	362 (30.94)	1,170	
**Gender**				0.351				1.000				0.181
Female	20,240 (69.94)	8,700 (30.06)	28,940		10,131 (70.03)	4,336 (29.97)	14,467		10,109 (69.85)	4,364 (30.15)	14,473	
Male	16 (61.54)	10 (39.46)	26		11 (68.75)	5 (31.25)	16		5 (50.00)	5 (50.00)	10	
**Grade**				**<0.001**				**<0.001**				**<0.001**
I	475 (87.80)	66 (12.20)	541		249 (89.89)	28 (10.11)	277		226 (85.61)	38 (14.39)	264	
II	3,546 (74.12)	1,238 (25.88)	4,784		1,796 (73.94)	633 (26.06)	2,429		1,750 (74.31)	605 (25.69)	2,355	
III	16,235 (68.67)	7,406 (31.33)	23,641		8,097 (68.75)	3,680 (31.25)	11,777		8,138 (68.59)	3,726 (31.41)	11,864	
**Laterality**				0.369				0.194				0.98
Left	10,316 (69.69)	4,486 (30.31)	14,802		5,163 (69.54)	2,261 (30.46)	7,424		5,153 (69.84)	2,225 (30.16)	7,378	
Right	9,940 (70.18)	4,224 (30.07)	14,164		4,979 (70.53)	2,080 (29.47)	7,059		4,961 (69.82)	2,144 (30.18)	7,105	
Location*				**<0.001**				**<0.001**				**<0.001**
Central	624 (60.94)	400 (39.06)	1,024		336 (63.16)	196 (36.84)	532		288 (58.54)	204 (41.46)	492	
Inner	4,943 (79.82)	1,250 (20.18)	6,193		2,458 (79.86)	620 (20.14)	3,078		2,485 (79.78)	630 (20.22)	3,115	
Outer	9,738 (66.46)	4,915 (33.54)	14,653		4,874 (66.52)	2,453 (33.48)	7,327		4,864 (66.39)	2,462 (33.61)	7,326	
Overlap	4,836 (70.27)	2,046 (29.73)	6,882		2,414 (70.22)	1,024 (29.78)	3,438		2,422 (70.33)	1,022 (29.67)	3,444	
Tail	115 (53.74)	99 (46.26)	214		60 (55.56)	48 (44.44)	108		55 (51.89)	51 (48.11)	106	
**Histological type**				**<0.001**				**<0.001**				**<0.001**
IDC	17,512 (69.57)	7,661 (30.43)	25,173		8,710 (69.50)	3,823 (30.50)	12,533		8,802 (69.64)	3,838 (30.36)	12,640	
ILC	141 (55.95)	111 (44.05)	252		82 (62.12)	50 (37.88)	132		59 (49.17)	61 (50.83)	120	
IDC/ILC	184 (56.62)	141 (43.38)	325		97 (58.08)	70 (41.92)	167		87 (55.06)	71 (44.94)	158	
Others	2,419 (75.22)	797 (24.78)	3,216		1,253 (75.89)	398 (24.11)	1,651		1,166 (74.50)	399 (25.50)	1,565	
**T stage**				**<0.001**				**<0.001**				**<0.001**
T1	10,553 (81.77)	2,353 (18.23)	12,906		5,261 (81.91)	1,162 (18.09)	6,423		5,292 (81.63)	1,191 (18.37)	6,483	
T2	8,330 (65.03)	4,479 (34.97)	12,809		4,145 (64.44)	2,287 (35.56)	6,432		4,185 (65.63)	2,192 (34.37)	6,377	
T3	1,066 (46.82)	1,211 (53.18)	2,277		573 (49.87)	576 (50.13)	1,149		493 (43.71)	635 (56.29)	1,128	
T4	307 (31.52)	667 (68.48)	974		163 (34.03)	316 (65.97)	479		144 (29.09)	351 (70.91)	495	

^#^American Indian/AK Native, Asian/Pacific Islander.

*Central, code C50.0 and C50.1; Inner, code C50.2 and C50.3; Outer, code C50.4 and C50.5; Tail, code C50.6; Overlap, code C50.8. From SEER Coding Guidelines Breast 2018 manual, coding guideline breast C500-C509.

IDC, invasive ductal carcinoma; IDC/ILC, Infiltrating duct and lobular carcinoma; ILC, invasive lobular carcinoma; LN, lymph nodes.

Bold value indicates statistical significance.

### Independent Predictors in Training Set

According to univariate Cox analysis, age, race, location, grade, histologic type, and T stage were significantly associated with the positive rate of lymph nodes (**Table S1**). These factors were included in multivariate logistic regression analysis (**Table 2**). The result confirmed that grade was not an independent predictor (P=0.421) and the others were statistically significant and independent predictors for lymph node status (P<0.05).

**Table 2:** Multivariate logistic regression analysis of possible factors independently predicting positive lymph nodes in the training cohort.

	Training cohort		
	OR	95%CI	P
**Age**			**<0.001**
<60	Ref.	Ref.	
≥60	0.85	0.79–0.92	
**Race**			**0.010**
White	Ref.	Ref.	
Black	1.22	1.11–1.33	
Others^#^	0.98	0.85–1.13	
**Grade**			0.421
I	0.38	0.25–0.57	
II	0.93	0.84–1.04	
III	Ref.	Ref.	
Location*			**<0.001**
Central	1.07	0.88–1.30	
Inner	0.51	0.46–0.56	
Outer	Ref.	Ref.	
Overlap	0.82	0.75–0.90	
Tail	1.60	1.07–2.38	
**Histological type**			**<0.001**
IDC	Ref.	Ref.	
ILC	1.66	1.20–2.31	
IDC/ILC	1.45	0.99–2.12	
Others	0.66	0.58–0.75	
**T stage**			**<0.001**
T1	Ref.	Ref.	
T2	2.40	2.21–2.61	
T3	4.28	3.74–4.90	
T4	8.60	7.02–10.53	

^#^American Indian/AK Native, Asian/Pacific Islander.

*Central, code C50.0 and C50.1; Inner, code C50.2 and C50.3; Outer, code C50.4 and C50.5; Tail, code C50.6; Overlap, code C50.8. From SEER Coding Guidelines Breast 2018 manual, coding guideline breast C500-C509.

CI, confidence interval; IDC, invasive ductal carcinoma; IDC/ILC, Infiltrating duct and lobular carcinoma; ILC, invasive lobular carcinoma; LN, lymph nodes; OR, odds ratio; Ref., Reference.

Bold value indicates statistical significance.

### Construction and Validation of the Nomogram

**Figure 1:**
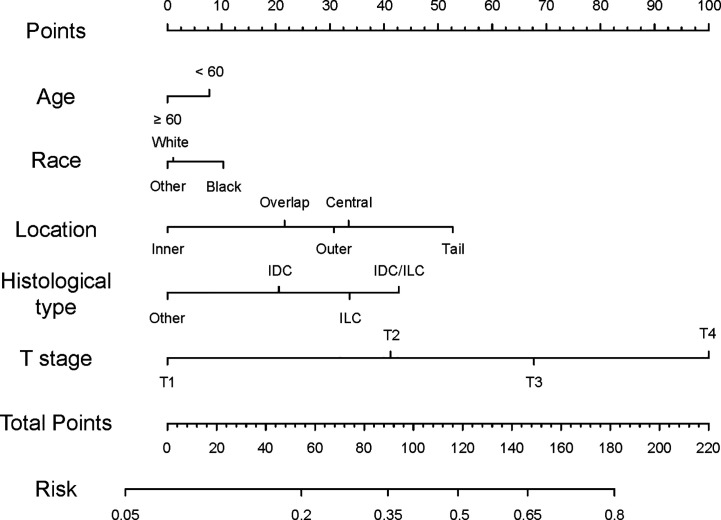
Nomogram predicting lymph node status of TNBC patients. Instructions for the nomogram: First, quantify each characteristic of the patient by drawing a vertical line from corresponding scale to the points scale. Then, sum all the points and draw a vertical line from the total points scale to the risk scale to obtain the probability of lymph node metastasis.

We established a nomogram based on significant and independent predictors determined by multivariate analysis (**Figure 1**), including age, race, location, histological type, and T stage. By adding up the scores of all the variables, the probability of a specific patient to have positive lymph nodes could be predicted. As we can see, younger black patients with T4 and IDC/ILC tumor at the axillary tail had highest scores, while elderly white cases with non-ILC or non-IDC, and T1 tumors had a lower risk of lymph node metastasis. The novel nomogram predicted the risk of positive lymph nodes between 0.05 and 0.8.

**Figure 2:**
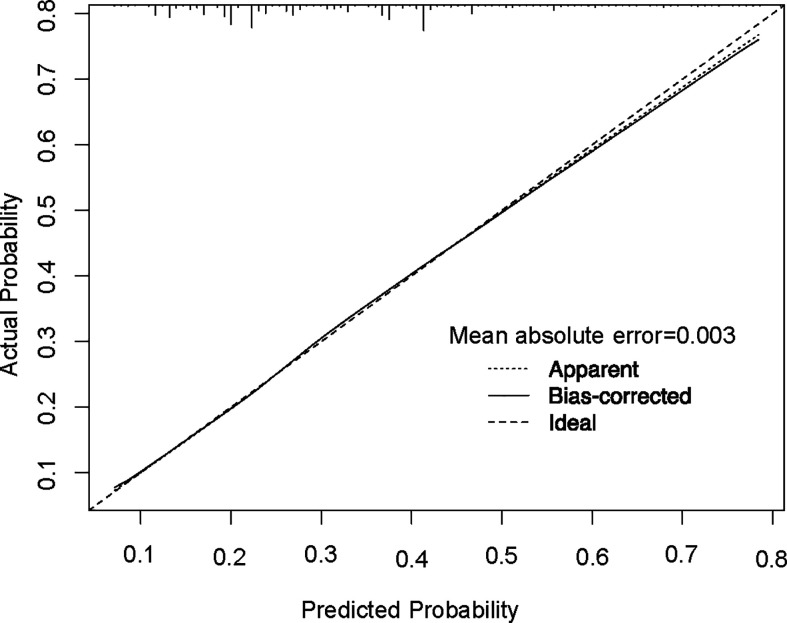
Calibration plot of the nomogram for predicting lymph node status. The performance is estimated by bootstrap 1,000 repetitions. The X-axis plots the nomogram-predicted survival; the Y-axis plots the actual survival.

**Figure 3:**
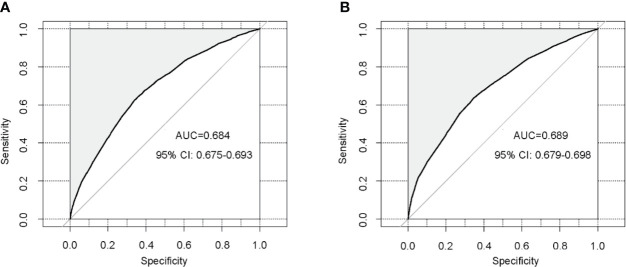
Validation of the nomogram using receiver operating characteristic curves. **(A)** Internal validation in the training cohort; **(B)** External validation in the validation cohort. AUC: area under curve; CI: confidence intervals.

In order to test the performance of the new nomogram, 1,000 bootstrap resampling was carried out for internal verification through the calibration chart in the training set (**Figure 2**). The calibration curve indicated a good calibration effect of the nomogram. The effectiveness of the nomogram for predicting lymph node status was further evaluated using ROC curves for the training (**Figure 3A**) and validation (**Figure 3B**) sets. In the training set, AUC was 0.684 (95%CI: 0.675–0.693), which is similar to the AUC observed in the validation set (0.689, 95%CI: 0.679–0.698). These results indicated that the nomogram is a useful predictor for lymph node status in TNBC.

### Risk Stratification by the Nomogram

The cut-off value of total scores for predicting lymph node status was determined by Youden’s index in the training set. Both the training and validation sets were subdivided into the low score groups (total points ≤ 82) and high score groups (total points>82), respectively. After applying the cut-off value to the training set, univariate analysis found a significant difference in the probability of lymph node metastasis between the high and low score groups (OR=3.24, 95%CI:3.03–3.49; P<0.001), consistent with the results obtained in the validation set (OR=3.30, 95%CI 3.07–3.56; P<0.001; **Table 3**).

**Table 3:** Univariate logistic regression analysis of total points in predicting positive lymph nodes in the training and validation cohorts.

Group^#^	Training cohort		P	Validation cohort		P
	OR	95%CI		OR	95%CI	
Low score	Ref.	Ref.	–	Ref.	Ref.	–
High score	3.24	3.03-3.49	**<0.001**	3.30	3.07–3.56	**<0.001**

**^#^**Low score, ≤82; high score,>82.

CI, confidence interval; OR, odds ratio; Ref., Reference.

Bold value indicates statistical significance.

## Discussion

In this study, the risk factors associated with lymph node metastasis in triple-negative breast cancer were determined, and a predictive model was developed by logistic regression, with a nomogram attached. We found that age, race, T stage, primary site, grade, and histological subtype were related to lymph node status by univariate logistic regression analysis. These variables were independent predictors of lymph node status confirmed by multivariate logistic regression except for grade. These factors were shown to be predictors of axillary lymph node metastasis. As shown above, the risk of lymph node metastasis was positively correlated with T stage. The increase in T stage was significantly associated with the risk of lymph node metastasis, which was previously reported (12). Young patients had higher odds of developing lymph node metastasis compared with older ones. Patients with the axillary tail as the primary site were more likely to have metastatic lymph nodes. These results indicate that the primary site of the tumor is important in predicting lymph node metastasis. It was also confirmed that the pathological type of ILC is more prone to lymph nodes metastasis. To validate the developed model, the bootstrapping method (1,000 times) was used. Moreover, the relatively high AUC of 0.684, also referred as concordance index in our study, confirmed the validation of this nomogram. Thus, this nomogram can be utilized by surgeons to more effectively counsel individual patients, thereby helping to personalize the surgical treatment of TNBC.

Previous studies have constructed nomograms to predict both sentinel and non-sentinel lymph node metastases in breast cancer, performing well in cohorts at different institutions (13–15). Several well-designed nomograms have been accepted worldwide, with some adopted by clinicians (16–20). For example, Hwang et al. incorporated sentinel lymph node metastasis size into a nomogram that accurately predicts the likelihood of having additional axillary metastasis (16). Nevertheless, these models only show limited performance in triple-negative breast cancer. For predicting non-sentinel lymph node metastasis in TNBC, some of these widely used nomograms are not much better than coin tossing, with AUCs around 0.55. It is noteworthy that such nomograms still work well in ER positive patients in the same institute (21). This phenomenon can be partly attributed to that rather than being a single subtype, triple-negative breast cancer is a general concept covering a group of diseases, with a variety in biological behavior, as well as great differences compared with other subtypes (22). To settle this, the cohort used to build a model should be large enough to cover each “subtype” of TNBC with an adequate number. SEER, a nationwide program covering nearly a quarter of the US population, is an optimal cohort for building such a model.

Apart from the excellent cohort as the data source for the nomogram, this model has other advantages. First, our research used the clinical information of TNBC patients to predict lymph node metastasis. Meanwhile, existing researches (10, 13) assessed TNBC at the genetic level, using IRGS to predict lymph node metastasis, and the obtained results were also good. However, clinical information is more intuitive to make decisions easily in clinic. Secondly, compared with the long and complicated formulas of Cox and logistic predictive models, nomograms, composed of several simple scaled parallel lines, provide a reliable prognostic information that is unique to a given patient.

Limited by the data and the characteristics of analysis, this study had some limitations. We were unable to obtain more information from the SEER database, including invasion of lymphatic or blood vessels, multifocality and even molecular biomarkers, which, if included, could improve the sensitivity and specificity of the present nomogram. In addition, as a retrospective study, selection or information bias was hardly avoidable. The main cohort in this study was the American population, and it is worth considering whether the results are applicable to other populations.

## Conclusion

In summary, a predictive nomogram for lymph node metastasis detection in TNBC patients was developed. Evaluating lymph node metastasis remains a major concern in the treatment and staging of breast cancer. The present findings reveal the features of lymph node metastasis in TNBC, providing a reference for future treatment which would take neoadjuvant chemotherapy and sentinel lymph node biopsy into consideration, eventually optimizing clinical diagnosis and treatment.

## Data Availability Statement

Publicly available datasets were analyzed in this study. This data can be found here: Surveillance, Epidemiology, and End Results (SEER) database (https://seer.cancer.gov/).

## Author Contributions

XC: conception of the work, data collection, data analysis and interpretation, drafting the article, critical revision of the article, and final approval of the version to be published. HZ and JH: conception of the work, critical revision of the article, and final approval of the version to be published. All authors contributed to the article and approved the submitted version.

## Funding

This work was supported by grants from the Training Plan of Excellent Talents of The First People’s Hospital of Shangqiu (SQFPH2019). The funder had no role in the study design, data collection and analysis, decision to publish, or preparation of the manuscript.

## Conflict of Interest

The authors declare that the research was conducted in the absence of any commercial or financial relationships that could be construed as a potential conflict of interest.

## Supplementary Material

Supplementary Table 1 Univariate logistic regression analysis of different variables in predicting positive lymph nodes in the training cohort. ^#^ American Indian/AK Native, Asian/Pacific Islander. * Central, code C50.0 and C50.1; Inner, code C50.2 and C50.3; Outer, code C50.4 and C50.5; Tail, code C50.6; Overlap, code C50.8. From SEER Coding Guidelines Breast 2018 manual, coding guideline breast C500-C509. CI, confidence interval; IDC, invasive ductal carcinoma; IDC/ILC, Infiltrating duct and lobular carcinoma; ILC, invasive lobular carcinoma; LN, lymph nodes; OR, odds ratio; Ref., Reference.

## References

[1] Siegel R Miller K Jemal A Cancer statistics, 2018. CA: Cancer J Clin (2018) 68:7–30. 10.3322/caac.21442 29313949

[2] DeSantis C Ma J Gaudet M Newman L Miller K Goding Sauer A Breast cancer statistics, 2019. CA: Cancer J Clin (2019) 69:438–51. 10.3322/caac.21583 31577379

[3] Pan K Zhang L Gerhard M Ma J Liu W Ulm K A large randomised controlled intervention trial to prevent gastric cancer by eradication of Helicobacter pylori in Linqu County, China: baseline results and factors affecting the eradication. Gut (2016) 65:9–18. 10.1136/gutjnl-2015-309197 25986943

[4] Barry P Vatsiou A Spiteri I Nichol D Cresswell G Acar A The Spatiotemporal Evolution of Lymph Node Spread in Early Breast Cancer. Clin Cancer Res an Off J Am Assoc Cancer Res (2018) 24:4763–70. 10.1158/1078-0432.Ccr-17-3374 PMC6296441 29891724

[5] Taylor K O’Keeffe S Britton P Wallis M Treece G Housden J Ultrasound elastography as an adjuvant to conventional ultrasound in the preoperative assessment of axillary lymph nodes in suspected breast cancer: a pilot study. Clin Radiol (2011) 66:1064–71. 10.1016/j.crad.2011.05.015 21835398

[6] Nathanson S Krag D Kuerer H Newman L Brown M Kerjaschki D Breast cancer metastasis through the lympho-vascular system. Clin Exp Metastasis (2018) 35:443–54. 10.1007/s10585-018-9902-1 29796854

[7] Morrow M Jagsi R McLeod M Shumway D Katz S Surgeon Attitudes Toward the Omission of Axillary Dissection in Early Breast Cancer. JAMA Oncol (2018) 4:1511–6. 10.1001/jamaoncol.2018.1908 PMC6248076 30003237

[8] Hindié E Groheux D Brenot-Rossi I Rubello D Moretti J Espié M The sentinel node procedure in breast cancer: nuclear medicine as the starting point. J Nuclear Med Off Publication Soc Nuclear Med (2011) 52:405–14. 10.2967/jnumed.110.081711 21321267

[9] Koca B Kuru B Ozen N Yoruker S Bek Y A breast cancer nomogram for prediction of non-sentinel node metastasis - validation of fourteen existing models. Asian Pacific J Cancer Prev APJCP (2014) 15:1481–8. 10.7314/apjcp.2014.15.3.1481 24606487

[10] Tan W Xie X Huang Z Chen L Tang W Zhu R Construction of an immune-related genes nomogram for the preoperative prediction of axillary lymph node metastasis in triple-negative breast cancer. Artif Cells Nanomed Biotechnol (2020) 48:288–97. 10.1080/21691401.2019.1703731 31858816

[11] Hosmer DW Hosmer T Le Cessie S Lemeshow S. A comparison of goodness-of-fit tests for the logistic regression model. Stat med (1997) 16:965–80. 10.1002/(sici)1097-0258(19970515)16:9<965::aid-sim509>3.0.co;2-o 9160492

[12] Rivadeneira DE Simmons RM Christos PJ Hanna K Daly JM Osborne MP Predictive factors associated with axillary lymph node metastases in T1a and T1b breast carcinomas: analysis in more than 900 patients. J Am Coll Surgeons (2000) 191:1–6. 10.1016/s1072-7515(00)00310-0 discussion -8. 10898177

[13] Wang N Yang Z Wang X Chen L Zhao H Cao W A mathematical prediction model incorporating molecular subtype for risk of non-sentinel lymph node metastasis in sentinel lymph node-positive breast cancer patients: a retrospective analysis and nomogram development. Breast Cancer (Tokyo Japan) (2018) 25:629–38. 10.1007/s12282-018-0863-7 29696563

[14] Tapia G Ying V Di Re A Stellin A Cai T Warrier S Predicting non-sentinel lymph node metastasis in Australian breast cancer patients: are the nomograms still useful in the post-Z0011 era? ANZ J Surg (2019) 89:712–7. 10.1111/ans.15173 31066184

[15] Han L Zhu Y Liu Z Yu T He C Jiang W Radiomic nomogram for prediction of axillary lymph node metastasis in breast cancer. Eur Radiol (2019) 29:3820–9. 10.1007/s00330-018-5981-2 30701328

[16] Mittendorf EA Hunt KK Boughey JC Bassett R Degnim AC Harrell R Incorporation of sentinel lymph node metastasis size into a nomogram predicting nonsentinel lymph node involvement in breast cancer patients with a positive sentinel lymph node. Ann Surg (2012) 255:109–15. 10.1097/SLA.0b013e318238f461 PMC4760742 22167004

[17] Vaysse C Sroussi J Mallon P Feron JG Rivain AL Ngo C Prediction of axillary lymph node status in male breast carcinoma. Ann Oncol Off J Eur Soc Med Oncol (2013) 24:370–6. 10.1093/annonc/mds283 23051951

[18] Park J Fey JV Naik AM Borgen PI Van Zee KJ Cody HS 3rd. A declining rate of completion axillary dissection in sentinel lymph node-positive breast cancer patients is associated with the use of a multivariate nomogram. Ann Surg (2007) 245:462–8. 10.1097/01.sla.0000250439.86020.85 PMC1877014 17435554

[19] Werkoff G Lambaudie E Fondrinier E Levêque J Marchal F Uzan M Prospective multicenter comparison of models to predict four or more involved axillary lymph nodes in patients with breast cancer with one to three metastatic sentinel lymph nodes. J Clin Oncol Off J Am Soc Clin Oncol (2009) 27:5707–12. 10.1200/jco.2009.21.9139 19826125

[20] Katz A Smith BL Golshan M Niemierko A Kobayashi W Raad RA Nomogram for the prediction of having four or more involved nodes for sentinel lymph node-positive breast cancer. J Clin Oncol Off J Am Soc Clin Oncol (2008) 26:2093–8. 10.1200/jco.2007.11.9479 18445838

[21] Ozbas S Ozmen V Igci A Muslumanoglu M Ozcinar B Balkan M Predicting the likelihood of nonsentinel lymph node metastases in triple negative breast cancer patients with a positive sentinel lymph node: Turkish Federation of Breast Disease Associations protocol MF09-01. Clin Breast Cancer (2012) 12:63–7. 10.1016/j.clbc.2011.07.004 22130034

[22] Bryan B Schnitt S Collins L Ductal carcinoma in situ with basal-like phenotype: a possible precursor to invasive basal-like breast cancer. Modern Pathol an Off J U States Can Acad Pathol Inc (2006) 19:617–21. 10.1038/modpathol.3800570 16528377

