# Supplementary material for: Nomogram for Predicting Lymph Node Involvement in Triple-Negative Breast Cancer
Source: Front Oncol. 2020 Dec 4;10:608334. doi: 10.3389/fonc.2020.608334 (PMC7747752; doi:10.3389/fonc.2020.608334)
Supplement: Supplementary Table 1 — Univariate logistic regression analysis of different variables in predicting positive lymph nodes in the training cohort. # American Indian/AK Native, Asian/Pacific Islander. * Central, code C50.0 and C50.1; Inner, code C50.2 and C50.3; Outer, code C50.4 and C50.5; Tail, code C50.6; Overlap, code C50.8. From SEER Coding Guidelines Breast 2018 manual, coding guideline breast C500-C509. CI, confidence interval; IDC, invasive ductal carcinoma; IDC/ILC, Infiltrating duct and lobular carcinoma; ILC, invasive lobular carcinoma; LN, lymph nodes; OR, odds ratio; Ref., Reference. [file Table_1.pdf]

SUPPLEMENTARY TABLE

TABLE S1 Univariate logistic regression analysis of different variables in predicting positive lymph nodes in the training cohort

	Training cohort		
	**OR**	**95%CI**	**P**
**Age**			**<0.001**
<60	Ref.	Ref.	
≥60	0.75	0.69-0.80	
**Race**			**<0.001**
White	Ref.	Ref.	
Black	1.29	1.18-1.41	
Others[#]	1.01	0.88-1.16	
**Grade**			**<0.001**
I	0.25	0.17-0.37	
II	0.78	0.70-0.86	
III	Ref.	Ref.	
**Location***			**<0.001**
Central	1.16	0.97-1.39	
Inner	0.50	0.45-0.55	
Outer	Ref.	Ref.	
Overlap	0.84	0.77-0.92	
Tail	1.59	1.08-2.33	
**Histological type**			**<0.001**
IDC	Ref.	Ref.	
ILC	1.64	1.21-2.24	
IDC/ILC	1.39	0.98-1.98	
Others	0.72	0.64-0.82	
**T stage**			**<0.001**
T1	Ref.	Ref.	
T2	2.50	2.30-2.71	
T3	4.55	3.99-5.19	
T4	8.78	7.19-10.71	

[#] American Indian/AK Native, Asian/Pacific Islander

* Central, code C50.0 and C50.1; Inner, code C50.2 and C50.3; Outer, code C50.4 and C50.5; Tail, code C50.6; Overlap, code C50.8. From SEER Coding Guidelines Breast 2018 manual, coding guideline breast C500-C509.

CI, confidence interval; IDC, invasive ductal carcinoma; IDC/ILC, Infiltrating duct and lobular carcinoma; ILC, invasive lobular carcinoma; LN, lymph nodes; OR, odds ratio; Ref., Reference.
